# Inflammation of Dentine or Tooth Bone

**Published:** 1853-10

**Authors:** 


### Dental Abstracts.

*Inflammation of Dentine or Tooth-Bone.*—An article on this subject is published in the Dental News-Letter, from the pen of Prof. Elisha Townsend, of Philadelphia, in the July No. of Vol. 6. He alludes to the low degree of vitality of tendons, hair and nails, and contends that "pain in inflamed bone and inflamed dentine must be allowed to prove the same point." That is vitality and distribution of nervous fibres through their structure, as well as through the tendons, which is proved in the latter by twisting, although they "may be cut, bruised, and even burnt without pain."

He accounts for the fact that a dead tooth is susceptible to impressions of heat or cold, by its being a better conductor than the living one.

In relation to the inflammation of the dentine he remarks: "But it is indeed enough that the teeth are parts of the living frame, subject to its diseases and united with it in functions, to establish the proposition on *a priori* principles of reasoning. The hair is alive, and the nails, for the difference between death and life even in these remotest territories of the animal organism is perfectly obvious; hair does not show its diseases by the clear signs that other parts do; that is, it does not become visibly inflamed or suffer pain, but it can die, and will indicate the fact in its own way. A finger nail that has lost its vitality is strongly marked by the fact, as a little attention will show. But the teeth exceed ordinary bones, I think, by the strength and clearness of their pathological conditions. Now, all these observations apply to diseases of those teeth, for which we resort to the remedy of excision of diseased matter and filling with gold, very clearly; and perhaps in more particulars than at present I have occasion to notice. Recollect that the dentine is capable of inflammation, of destructive inflammation. If the symptoms are less obvious, they are nevertheless as positive. They are modified by the character of the organization, and especially by the texture of the part. The vessels are too firmly impacted in earthy matter, of the greatest density that occurs any where in the living body, to allow of their enlargement to the extent that could make such enlargement visible. Hence, we see no redness, and no swelling of the mass in which they are situated. The pain is decided, for that is the tenderness to the touch which is felt when the diseased surface, the bony ulcer, or ulcer in the dentine, is touched with an instrument or other irritant; it is the inflammation, or exalted action in the vascular structure which occasions this tenderness. If you boldly cut below and beyond this inflamed substance, you will reach the healthy part which, uninflamed, bears touching freely, and all the pressure of filling besides; when this is thoroughly done, I mean the tender portion perfectly removed, no pressure that can be applied by the operator in packing the filling, can artificially compress the nervous fibre so as to give pain: for the closeness of its earthy particles is greater than any power the dentist's hand can equal. Again, the metallic filling is not an irritant, and will not awaken the sensibility. If, however, the gold is not well consolidated, and the saliva is admitted around its edges, the mischief will again begin, and this is an additional reason for avoiding as much as possible the admission of saliva during the time of plugging. Simple water is by no means so injurious; nay, the pressure employed *may*, perchance, wholly exclude it, but the water of saliva contains mucus and salts, likely to adhere in such case, and do the mischief which we dread. We generally find the most sensitive portions of the tooth in the part immediately subjacent to the enamel, at the very extremity then of the nervous life of the tooth, at the extremities or ends of the nervous fibres. We often find upon opening the pulp cavity of a tooth in health, that the main trunk of the nerve is even less tender, and less complained of by the patient, though we extirpate it with an instrument, than the edges of the cavity at the extremities of the nervous life of the dentine.

"Now, it is known, as a general law of the living organism, that the special function of a part is seated in the extremities. In the arteries all the actions of nutrition are certainly performed at the ultimate points of the minutest vessels. Again, in the reparation of parts destroyed by ulceration or external violence, the granulations are produced from the extremities of the vessels, for they are deposited upon the surface of the wound. A blush upon the cheek, and every in stance of efflorescence, however induced, are clear examples of activity in the capillaries, independent of, and in the absence of all excitement in the general circulation. This shows an independent capability of action in the radical tubes, and sustains the assertion that the vitality of the vessels is eminently in their extremities; the larger branches are only so many conduits, or conveyors of the vital fluid, and are not, as they need not be, endowed with any greater sensibility or functional activity, than such ancillary office demands. Of the nerves the same thing is true, as in those devoted to the external senses of vision and smell; here the special power of the nerve is found not *at all* in the trunk, but in the expanded a nd ramified extremity. The office of the trunks and branches of considerable size—it may be said of all sizes, which are easily exhibited by dissection—is merely that of conveying impressions from extremities, where they are received, to the brain, and conversely, the impulse of the brain outward to the organs of motion, &c. These larger branches and their common stems are not only destitute of the specific functions belonging to their extremest fibrillæ, but they have very little, if any thing more than the common sensibility to violence and irritants that belongs to all the soft parts of the body. In their healthy state, they bear the knife in amputation, as well as the muscular fibres do; indeed, the principal pain in ordinary surgical cases, where the incisions are made in healthy textures, is felt in the skin, where of necessity the extreme fibrils of the nerves only are found. It must be observed as a general idea governing all indications from facts as they occur to observation, that the sensibility is proportioned and adjusted to their uses."

